# Draft Genome Sequence of Klebsiella pneumoniae subsp. *pneumoniae* CCI2, Isolated from Leaf Soil

**DOI:** 10.1128/MRA.00343-21

**Published:** 2021-06-24

**Authors:** Hironaga Akita, Yuya Itoiri, Noriyo Takeda, Zen-ichiro Kimura, Hiroyuki Inoue, Akinori Matsushika

**Affiliations:** a Research Institute for Sustainable Chemistry, National Institute of Advanced Industrial Science and Technology (AIST), Higashi-Hiroshima, Hiroshima, Japan; b Department of Civil and Environmental Engineering, National Institute of Technology, Kure College, Kure, Hiroshima, Japan; c Graduate School of Integrated Sciences for Life, Hiroshima University, Higashi-Hiroshima, Hiroshima, Japan; University of Maryland School of Medicine

## Abstract

Klebsiella pneumoniae subsp. *pneumoniae* CCI2 was isolated from leaf soil collected in Hiroshima Prefecture, Japan. The draft genome sequence comprises 78 contigs and contains 5,075,115 bp with a G+C content of 57.7%.

## ANNOUNCEMENT

Strain CCI2 (strain number HUT-8144), which was isolated from leaf soil in Higashi-Hiroshima (GPS coordinates: 34.40535090699955, 132.751337987168), Hiroshima, Japan, using a 1.5% agar plate (1), had a low nutrient requirement and was able to grow under poor-nutrient conditions. Using nutrient broth (Kyokuto) as growth medium, the effects of culture temperature and pH were examined (1), and this strain showed a wide range of viable temperatures (10 to 45°C) and pHs (4.0 to 11.0). Moreover, the strain was tolerant to 8.0% (wt/vol) NaCl. Thus, strain CCI2 may have potential as a host for fermentation for the production of useful substances. To determine the phylogenetic position of strain CCI2, we performed 16S rRNA gene and draft genome sequencing as well as average nucleotide identity (ANI) value analysis.

To yield the genomic DNA, strain CCI2 was cultured at 37°C for 12 h in nutrient broth. After the genomic DNA from the culture was extracted using an illustra bacteria genomicPrep mini spin kit (GE Healthcare), the concentration and purity were measured using a Quant-iT double-stranded DNA (dsDNA) assay kit (Invitrogen) and a NanoDrop ND-1000 spectrophotometer (Thermo Fisher Scientific), respectively. Using the genomic DNA as the template, the 16S rRNA gene was amplified using KOD Plus (Toyobo) with the universal bacterial primers 27f and 1391r. The homology search was carried out using the NCBI BLAST program. The sequence libraries for genome sequencing were prepared using a Nextera XT DNA library preparation kit (Illumina) according to the manufacturer’s instructions. The resulting libraries were sequenced using a MiSeq sequencer (Illumina) with a MiSeq reagent kit v3 (Illumina). Default parameters were used for all software unless otherwise specified. The *de novo* assembly and the quality control were carried out using A5-miseq v.20160825 (2). Genome annotation and the detection of tRNA and rRNA genes were carried out using Prokka v.1.14.0 (3). The ANI value was calculated using the ANI algorithm (4) implemented within OrthoANIu tools (5).

The 16S rRNA gene sequence (accession number LC589705) of strain CCI2 showed 99.3%, 98.7%, and 98.2% similarities to Klebsiella variicola subsp. *variicola* F2R9^T^ (6), Klebsiella quasipneumoniae subsp. *quasipneumoniae* 01A030^T^ (7), and Klebsiella pneumoniae subsp. *ozaenae* ATCC 11296^T^ (8), respectively. Based on the sequence similarity, strain CCI2 could be a novel species. Subsequently, strain CCI2 was subjected to genome sequencing. The raw data included 10,116,496 paired-end reads with a length of 301 bp that were used for the assembly. The draft genome sequence of strain CCI2 consists of 78 contigs containing 5,075,115 bp with a G+C content of 57.7% and coverage of 525-fold. The largest contig is 513,002 bp, and the *N*_50_ contig size is 178,989 bp. Genome annotation revealed 4,677 predicted coding sequences, 81 tRNA genes, and 18 rRNA genes. The ANI value between strain CCI2 and its closest relative, Klebsiella pneumoniae subsp. *pneumoniae* ATCC 13883^T^, was 99.2%, which exceeded the cutoff value of 98% for prokaryotic subspecies delineation (9, 10). Thus, we identified strain CCI2 as K. pneumoniae subsp. *pneumoniae* CCI2.

### Data availability.

The draft genome sequence of K. pneumoniae subsp. *pneumoniae* CCI2 has been deposited in DDBJ/EMBL/GenBank databases under accession numbers BNJO01000001 to BNJO01000078. The raw sequence reads have been deposited in the DDBJ database under BioProject number PRJDB10686, BioSample number SAMD00253847, and DRA number DRA011085.
